# Child-OIDP index in Brazil: Cross-cultural adaptation and validation

**DOI:** 10.1186/1477-7525-6-68

**Published:** 2008-09-15

**Authors:** Rodolfo AL Castro, Maria IS Cortes, Anna T Leão, Margareth C Portela, Ivete PR Souza, Georgios Tsakos, Wagner Marcenes, Aubrey Sheiham

**Affiliations:** 1 National School of Public Health, Oswaldo Cruz Foundation, Rua Leopoldo Bulhões, 1480/724, Manguinhos, Rio de Janeiro, 21031-210, Brazil; 2 School of Dentistry, Pontifical Catholic University of Minas Gerais, Avenida Dom Jose Gaspar, 500, Prédio 46, Coração Eucarístico, Belo Horizonte, 35588-000, Brazil; 3 School of Dentistry, Federal University of Rio de Janeiro, Avenida Brigadeiro Trompovsky s/n, Rio de Janeiro, 21941-590, Brazil; 4 University College London, 1-19 Torrington Place, London, WC1E 6BT, UK; 5 Barts and the London School of Medicine and Dentistry, Institute of Dentistry, Turner Street, London, E1 2AD, UK

## Abstract

**Background:**

Oral health-related quality of life (OHRQoL) measures are being increasingly used to introduce dimensions excluded by normative measures. Consequently, there is a need for an index which evaluates children's OHRQoL validated for Brazilian population, useful for oral health needs assessments and for the evaluation of oral health programs, services and technologies. The aim of this study was to do a cross-cultural adaptation of the Child Oral Impacts on Daily Performances (Child-OIDP) index, and assess its reliability and validity for application among Brazilian children between the ages of eleven and fourteen.

**Methods:**

For cross-cultural adaptation, a translation/back-translation method integrated with expert panel reviews was applied. A total of 342 students from four public schools took part of the study.

**Results:**

Overall, 80.7% of the sample reported at least one oral impact in the last three months. Cronbach's alpha was 0.63, the weighted kappa 0.76, and the intraclass correlation coefficient (ICC) 0.79. The index had a significant association with self-reported health measurements (self-rated oral health, satisfaction with oral health, perceived dental treatment needs, self-rated general health; all p < 0.01).

**Conclusion:**

It was concluded that the Child-OIDP index is a measure of oral health-related quality of life that can be applied to Brazilian children.

## Background

The World Health Organization (WHO) defines health as a "state of complete physical, mental and social well-being and not merely the absence of disease or infirmity" [1]. Based on this concept, measuring health should not be confined to the use of exclusively clinical normative indicators. Health-related quality of life (HRQoL) measures are being used nowadays to evaluate dimensions of health, such as psychological and social aspects, that are not assessed by other measures. HRQoL measures can be categorized as: generic or specific. The generic measures are used to evaluate the impact of general health problems on quality of life. The specific measures focus on the repercussions of particular health conditions, health problems or treatments on the quality of life [2].

Oral health-related quality of life (OHRQoL) indices have a specific application in the evaluation of the impacts of oral problems on daily activities. These indices are most commonly used for adults or elderly populations. Some authors have adapted and applied instruments developed for adults to children and adolescents [3,4]. However, there is a trend to generate specific indices which cater for the needs of younger populations [5,6]. One of the measures developed specifically for children is the Child Oral Impacts on Daily Performances (Child-OIDP) index. Child-OIDP was developed in English, then validated in Thailand, and more recently in other countries [7-10]. The objective of this index is to measure the impacts of oral health problems on daily activities commonly performed by children. It comprises dimensions not tapped by clinical measures, such as functional, psychological and social limitations. The index has applications in public health for the assessment of oral health needs and can be a valuable indicator for the evaluation of oral health programs [11].

The psychometric properties of the Child-OIDP have been successfully assessed in several countries with different cultures and languages, such as Thai, French, English, Spanish and Kiswahili, but not in Portuguese. The availability of multi-lingual versions of instruments is important for epidemiological research. The aim of this study was to carry out a cross-cultural adaptation of the Child-OIDP index and to assess its reliability and validity for application among Brazilian children between the ages of eleven and fourteen.

## Methods

### Study design and ethical considerations

The methodology emphasises the cross-cultural adaptation of the Child-OIDP and its psychometric testing for test-retest reliability, internal consistency and construct validity. Informed consent was sought and obtained from the parents of the participants. The research protocol was approved by the Ethics Committee of the National School of Public Health of Brazil (approval number: 04/05).

### Description of the index

For the application of the Child-OIDP the children were initially asked to record all oral health related problems they have experienced in the past three months (Table 1). This was done in small groups, in order to reduce time. Then, data were collected on the impacts of oral problems, through face-to-face interviews, considering eight common daily performances. These performances are: eating, speaking, cleaning mouth, sleeping, emotional status, smiling, studying, and social contact (Table 1).

**Table 1:** Items and modifications from the original questionnaire (changes are in bold italics)

**First part of the instrument**	
**List of problems of the original Child-OIDP**	**List of problems of the Brazilian Child-OIDP (translated)**
Toothache	Toothache
Sensitive tooth	Sensitive ***teeth *(*when you eat or drink: sweets, hot food such as milk or coffee and cold food such as ice cream*)**
Tooth decay, hole in tooth	Tooth decay, hole in tooth
Exfoliating primary tooth	***mobile milk teeth***
Tooth space (due to an non-erupted permanent tooth)	Tooth space (due to an non-erupted permanent tooth)
Fractured permanent tooth	***broken permanent (new or definitive) tooth***
Colour of tooth	Colour of tooth ***(darker or more yellow in color, or stained)***
Shape or size of tooth	shape or size of tooth ***(abnormally sized or shaped tooth, or larger or smaller than the other teeth)***
Position of tooth (e.g. crooked or projecting, gap)	tooth position (***crowded***, crooked, ***separated***, or ***protruding teeth***)
Bleeding gum	bleeding of the gums (***when brushing teeth***)
Swollen gum	swollen gums (***inflamed or very red gums***)
Calculus	***tartar***
Oral ulcers	oral ***wounds***
Bad breath	Bad breath (***bafo: a popular term in Portuguese with no translation to english***)
Erupting permanent tooth	Erupting permanent tooth
Missing permanent tooth	Missing, ***lost, or extracted ***permanent tooth
Other (specify)	***Others. Which?***

**Second part of the instrument**	
**Performances of the original Child-OIDP**	**Performances of the Brazilian Child-OIDP (translated)**

Eating food (e.g. meal, ice-cream)	Eating food (e.g. meal, ice-cream)
Speaking clearly	Speaking clearly
Cleaning your mouth (e.g. rinsing your mouth, brushing your teeth)	Cleaning your mouth (e.g. rinsing your mouth, brushing your teeth)
Relaxing (including sleeping)	***Sleeping***
Maintaining your usual emotional state without being irritable	Maintaining your emotional state (***mood***) without becoming irritated ***or stressed***
Smiling, laughing and showing your teeth without embarrassment	Smiling, laughing and showing your teeth without embarrassment
Carrying out your schoolwork (e.g. going to school, learning in class, doing homework)	Carrying out your schoolwork (e.g. going to school, learning in class, doing homework)
Contact with people (e.g. going out with friend, going to a friend's house)	Contact with people (e.g. going out with friend, going to a friend's house)

In the event that a child reported an impact on their performance of these eight daily performances, the child responded to questions about the severity and frequency of the specific impact; a score from 0 to 3 is given to rate each of these characteristics. When no impact had been reported, the child received a score of zero. The calculation of the index involves the multiplication of the severity and frequency of each performance. A sum is made of the values obtained for the eight performances, resulting in a number from 0 to 72, which is divided by 72 and then multiplied by 100, so that the final Child-OIDP score varies from 0 to 100. A more detailed description of the index can be obtained in the development paper of the Child-OIDP [5].

### Cross-cultural adaptation

The methods used to translate the questions in the Child-OIDP index to Portuguese and to adapt the index to the Brazilian culture followed published guidelines [12]. The process of cross-cultural adaptation was conducted in Rio de Janeiro and involved several steps: translation from English to Portuguese; first meeting of the expert panel to produce the first Portuguese version; pilot-testing in a focus group of children; second meeting of the expert panel to produce a new consensus version; back-translation to English; re-evaluation by the expert panel members and by the authors of the original Child-OIDP.

The Child-OIDP was translated from English to Portuguese by three native Portuguese-speaking professional translators. Two of the three translators were unaware of the concepts used and of the objectives of the study. These three versions of the index were assessed by an expert panel involving five specialists: two specialists in quality of life measures, two experienced pediatric dentists, and one specialist in Health Policy and Administration. Collectively, this team compiled a single version of Child-OIDP. This version was assessed for understanding and to adjust the instrument's terminology in a focus group study composed of ten 11–14 years-olds. Additionally, the questionnaire was applied to a convenience sample of thirteen 11–14 year-olds in a public school in Nova Iguaçu, Rio de Janeiro to check the operational performance. The expert panel reviewed the results from the focus groups and produced the first consensus version of the Brazilian Child-OIDP.

To test the cross-cultural adaptation, the Brazilian Portuguese consensus version of the index was back-translated to English by two independent native English-speaking professional translators. The index was then re-evaluated for adequacy by the members of the expert panel and by the authors of the original version of the Child-OIDP.

### Reliability and validation studies

The Brazilian Child-OIDP [see Additional file 1] was administered to 342 children between the ages of 11 and 14 in four different public schools located in the southeast of Brazil. Two of the schools were located in the city of Rio de Janeiro (n = 203) and two in Belo Horizonte (n = 139). All were public schools. In Rio de Janeiro, one school was in a deprived area and the other in a semi-deprived area; in Belo Horizonte, both schools were in a semi-deprived area. Children were randomly selected from official school registries.

Two trained dentists conducted the interviews. Instructions for the interview were given by the authors of the original questionnaire. The first part of the Child-OIDP assessed common oral health problems, and was conducted in small groups of six children. Each child answered the questionnaire without communicating with each other. The second part assessed impacts of oral health problems in a face-to-face interview performed with each child individually. Also, it included questions on self-rated oral and general health.

For test/retest reliability measurements, 20 children received an additional interview with the index within one-week interval of the first administration. Reliability was tested using the weighted-kappa and the intraclass correlation coefficient (ICC).

In addition to the Child-OIDP, the interview also contained the following questions (all with 3-point ordinal scales) that were used for the assessment of construct validity: self-rated oral health (answers ranging from "good" to "poor"), satisfaction with oral health (answers ranging from "not at all" to "very satisfied"), perceived oral treatment needs ("yes", "do not know", "no"), and self-rated general health (answers ranging from "good" to "poor").

### Data analysis

Data were entered into Epi Info (version 3.4.3), and analyses were performed using SAS statistical package (version 9.1). Reliability testing referred to internal consistency and test-retest reliability. Internal consistency was evaluated using the Cronbach's alpha, alpha if item deleted, and inter-item and item-total correlation coefficients with Pearson correlation coefficients (PCC). Test-retest reliability was determined by using: a) weighted Kappa, with the Child-OIDP score categorised into five groups, and b) ICC for the Child-OIDP score.

To establish construct validity, the Brazilian Child-OIDP score was compared between the different groups of other subjective oral and general health status variables (self-rated oral health, satisfaction with oral health, perceived oral treatment needs, and self-rated general health), through the use of the Kruskal-Wallis test.

## Results

To accomplish an accurate cross-cultural adaptation of the index, some words had to be modified from the original version. The decision to modify the index was made collectively by the expert panel, using notes and data obtained in the pilot testing. The experience of professionals of pediatric dentistry in the expert panel that knew the terms used by children when referring to oral health and problems was important for the modification process. The modifications did not affect the content of the index but aimed to facilitate comprehension and ease of administration in the culturally specific context. They varied from broader issues, such as the use of pictures and examples in answering options (pictures of a facial scale were used for all children to help them decide on the severity of an impact), to very detailed modifications, such as the choice of the appropriate marking symbol. The choice of alternatives in the self-administered first part of the index was supposed to be marked with a symbol (v), in Brazil it is more common to use an "X" between two parenthesis than using the suggested mark, so the "X" was adopted in the Brazilian version. In addition, some terms used in the questionnaire were replaced for words more common in Brazil. Also, examples were included after "teeth/mouth problems", in order to make the content more specific and facilitate understanding (Table 1). To help children's comprehension of the severity of the impact, a facial scale with three different expressions was added to the arrows presented in the original version of the Child-OIDP. When asking about the frequency of a problem a navigation question was inserted: "Did it happen one or more times a month, or less than once a month?" to decide if the problem has happened on a regular basis or not. If the problem was on a regular basis, the child was asked about the number of times it occurred. If it did not happen on a regular basis, the next question was about on how many days in total it happened. One of the performances was modified from "relaxing (including sleeping)" to "sleeping", since it was observed in the pilot testing that the children did not use the term relaxing.

A total of 540 children were invited to participate in the validation study and 342 parents signed the informed consent, resulting in a response rate of 63.3%. The mean age of the subjects was 12.8 (sd: ± 1.1), and the median was 12.7. There were 172 (50.3%) girls and 170 (49.7%) boys in the sample.

The sample reported high levels of perceived oral problems. The most prevalent perceived oral problem reported in the first step of the Child-OIDP was sensitive teeth (63.2%) followed by tooth color (42.4%) (Table 2). Overall, 80.7% of the sample reported at least one oral impact in the last three months. The performances with the highest frequencies impacts were "eating" (59.4%), "emotional status" (33.6%), "cleaning mouth" (33.3%) and "smiling" (21.3%), while the performance with the lowest impact was "studying" (6.7%) (Table 3). The mean Child-OIDP score was 9.2 (sd: ± 10.1), quartile 75%: 13.9, median: 5.5, and, quartile 25%: 1.4. When the index was analyzed by performances, eating had the highest mean impact score (Table 3).

**Table 2:** Prevalence of perceived oral problems in 11–14 year old Brazilian children (n = 342)

**List of common oral problems**	**Children with the problem % (n)**
Sensitive tooth	63.2 (216)
Tooth color	42.4 (145)
Bleeding gums	36.8 (126)
Toothache	35.4 (121)
Dental caries	32.7 (112)
Position of teeth	32.2 (110)
Tooth shape or size	30.4 (104)
Erupting permanent tooth	25.4 (87)
Wounds	22.8 (78)
Bad breath	17.3 (59)
Space between teeth	16.1 (55)
Exfoliating primary teeth	14.6 (50)
Swollen gums	12.0 (41)
Broken permanent tooth	9.6 (33)
Tartar (calculus)	7.0 (24)
Missing permanent tooth	6.7 (23)
Deformed mouth or face	0.9 (3)
Other problems	2.3 (8)

**Table 3:** Prevalence of oral impacts on daily performances (Child-OIDP) in 11–14 year old Brazilian children

**Performances**	**Percentage of children with impact on performance (n = 342)**	**Mean Child-OIDP (± SD) on each performance (0 to 100)**
Eating	59.4	21.4 (25.9)
Speaking	14.0	5.6 (17.3)
Cleaning mouth	33.3	12.5 (23.1)
Sleeping	10.5	3.0 (11.3)
Smiling	21.3	7.7 (18.2)
Emotional status	33.6	15.7 (28.9)
Studying	6.7	2.6 (11.7)
Social contact	12.3	4.9 (16.1)
At least one of above	80.7	-

The test-retest reliability of the index using weighted-kappa for Child-OIDP categories was 0.76 and the average measure ICC for the Child-OIDP score was 0.79.

The internal consistency analysis of the Child-OIDP resulted in a standardized Cronbach's alpha of 0.63. There were no negative correlation coefficients when the inter-item correlation was done using PCC (Table 4). Alpha value decreased when any item was deleted. Considering item-total correlations, all items were above 0.20 (Table 5).

**Table 4:** Pearson correlation coefficients of performances of Child-OIDP index (n = 342)

	**Eating**	**Speaking**	**Cleaning Mouth**	**Sleeping**	**Smiling**	**Emotional status**	**Studying**	**Social Contact**
**Eating**	1							
**Speaking**	0.12^b^	1						
**Cleaning mouth**	0.30^a^	0.10^c^	1					
**Sleeping**	0.17^a^	0.18^a^	0.10^c^	1				
**Smiling**	0.23^a^	0.17^a^	0.32^a^	0.01^c^	1			
**Emotional status**	0.04^c^	0.17^a^	0.20^a^	0.05^c^	0.25^a^	1		
**Studying**	0.18^a^	0.14^b^	0.15^a^	0.29^a^	0.21^a^	0.00^c^	1	
**Social contact**	0.08^c^	0.23^a^	0.20^a^	0.07^c^	0.21^a^	0.24^a^	0.25^a^	1

^a ^p < 0.01

^b ^p < 0.05

^c ^Not significant

**Table 5:** Standardised Cronbach's alpha, item-total correlation and alpha with deleted items

**Standardised Cronbach's alpha**		0.63
	**Correlation with total**	**Alpha if deleted**

**Eating**	0.30	0.59
**Speaking**	0.29	0.59
**Cleaning mouth**	0.37	0.57
**Sleeping**	0.23	0.61
**Smiling**	0.38	0.56
**Emotional status**	0.25	0.60
**Studying**	0.33	0.58
**Social contact**	0.35	0.57

The relationship between the Child-OIDP score and the self-rated measures demonstrated that children with perceptions of poor oral health had a higher median score of the index (16.7) than children that evaluated their oral health as good (1.4). Similarly, children who were more satisfied with their oral health had a lower median Child-OIDP score. The perception of the presence of oral treatment needs and the poor self-rated general health was related with higher Child-OIDP index (Table 6). The results suggest that children perceived the "do not know" answering option as "I am not sure" when asked about the perceived oral treatment needs. So it is considered as an intermediate answer between "yes" and "no". There was a clear trend in all the responses, revealing a gradual increase in oral impacts with worsening subjective perceptions.

**Table 6:** Construct Validity: Child-OIDP score and self-rated measures of oral health (n = 342)

**Self-rated oral health measures**	**Child-OIDP Score**		**Kruskal-Wallis test for association between Child-OIDP and oral health measure**
		
	**Median**	**Mean (SD)**	
**Perceived oral health**			
1. Poor (n = 40)	16.7	17.8 (12.0)	p < 0.0001
2. Regular (n = 223)	6.9	8.9 (9.7)	
3. Good (n = 79)	1.4	5.5 (8.1)	
			
**Satisfaction with oral health**			
1. Not at all (n = 69)	13.9	15.5 (11.3)	p < 0.0001
2. Regular (n = 170)	5.5	8.0 (8.8)	
3. Very satisfied (n = 103)	2.8	6.8 (9.7)	
			
**Perceived oral treatment needs**			
1. Yes (n = 221)	8.3	11.1 (10.7)	p < 0.0001
2. Do not know (n = 41)	4.2	7.9 (9.0)	
3. No (n = 80)	1.4	3.5 (6.2)	
			
**Self-rated general health**			
1. Poor (n = 16)	11.8	17.4 (13.7)	p < 0.01
2. Regular (n = 86)	8.3	10.7 (11.0)	
3. Good (n = 240)	4.9	8.1 (9.4)	

## Discussion

The main contribution of this study was to rigourously adapt the Child-OIDP index for Brazilian children aged 11–14 years and successfully assess its psychometric properties in a sample drawn from two culturally different areas in Brazil. On the other hand, the following limitations should be pointed out: only public schools were included, a convenience rather than a random sampling approach was adopted, and the response rate was moderate.

The methods applied in the cross-cultural adaptation followed guidelines previously used in other validation studies [12] and assured the equivalence of the original and adapted versions. Although word modifications were made to take into account social and cultural differences, during this process, much care was taken to ensure that linguistic equivalence was achieved. Brazil has a continental dimension, with regional cultural differences. However, due to the fact that this study included two separate cities in different states, the predicted applicability of the Brazilian Child-OIDP may be considered nation-wide.

Test-retest reliability, evaluated using the Kappa and ICC, was very good and shows that the index is a stable measure. This result is comparable to other validation studies of the Child-OIDP [7-10]. As this index can be applied by any trained person, and not only a dentist, it can be used in public health programs as a sociodental indicator of oral health [11].

The internal consistency of the index, measured by the Cronbach's alpha coefficient, despite not being satisfactory based on the criteria that defines a cut-off of 0.7 for adequate consistency, was considered comparable to other results obtained when validating the Child-OIDP in other countries [7-9]. Quality of life is a multidimensional concept. Therefore, any measure of health-related impacts on daily life, including the Child-OIDP, needs to contain various dimensions. This may explain why the Child-OIDP index, considering all the items, did not reach an alpha of 0.7 or above, and yet was judged satisfactory. Moreover, when any of the items were deleted the alpha value decreased, hence providing evidence that all the items are important to the establishment of the index. A comprehensive evaluation of the validity of the Child-OIDP conducted in Peru reported that the small number of items present in the index results in a lower alpha [9]. Clearly, the value of alpha is based on the correlation between items and the number of items in a scale, with scales with fewer items tending to have lower alpha values [13-15]. As the Child-OIDP index is aimed to be a brief and cost-effective measure with high applicability in public health and needs assessment, it assesses oral impacts in relation to eight independent daily performances. Therefore, there is no merit in increasing the number of items, with the aim of achieving higher alpha values, as this will negatively affect the applicability of the measure. A relatively lower alpha value may be, to a certain extent, an inherent attribute of a brief and practical OHRQoL measure that can be used for needs assessment in a population [7].

Previous studies have adapted and applied sociodental indices developed for adults in adolescents in Uganda and in Brazil [3,4]. The present work validates an instrument specifically constructed for children. Anguita et al [16] concluded that the adaptation of an instrument is preferable to the development of a new one. Developing a new instrument can be complex; the adapted version can be as valid and reliable as the original; and, the presence of an instrument of reference helps investigations where various countries take part, by allowing for direct comparability of findings.

The prevalence of impacts observed in Brazil (80.7%) was comparable to those found in other countries where the Child-OIDP was adapted and applied: Thailand (89.8%), France (73.2%), Peru (82.0%) [17,7,18]. However, it was higher than in England (40.4%) and Tanzania (28.6%) [8,10]. In relation to the most prevalent oral impacts, eating and emotional status were the two performances mostly affected in Brazil, while in France and England cleaning mouth was the second most affected performance [8,9]. Eating was the most affected performance in all studies using Child-OIDP in a general population. Concerning the perceived oral problems, sensitive tooth and tooth color were the most commonly reported by the Brazilian children while in France the problems mentioned were position of teeth and wounds [7].

In the evaluation of the construct validity of the Child-OIDP index, the score increased progressively, indicating worse oral health-related quality of life, as the children's self-rated oral health status, satisfaction and treatment needs, as well as self-rated general health, changed from healthy to unhealthy. This consistent pattern throughout the construct validity testing is an interesting and strong finding, because rather than merely observing a difference in Child-OIDP scores between the worse off and the rest of the population, there were gradual trends in all aforementioned associations, therefore highlighting the close relationship between oral health-related quality of life and other subjective measures of oral and general health. These differences were statistically significant for all variables measured.

## Conclusion and recommendations for future research

It was concluded that the Child-OIDP index is a measure of oral health-related quality of life that can be applied to Brazilian children.

Future studies should be conducted on the Child-OIDP index to fully evaluate its psychometric properties in a population based epidemiological study. Its sensitivity to change should also be established, so that it can be considered for clinical trials to assess the effect of treatment on quality of life. Finally, the index can be used to assess the relationship of oral impacts and quality of life with clinical dental status and also contribute to assessing the dental treatment needs of children.

## Competing interests

The authors declare that they have no competing interests.

## Authors' contributions

RALC contributed with conception, design, acquisition of data, analysis, interpretation of data, draft and revision of the manuscript. MISC contributed with conception, design, acquisition of data, interpretation of data and revision of the manuscript. ATL and MCP contributed with conception, design, acquisition of data, analysis, interpretation of data and revision of the manuscript. IPRS, GT, WM and AS contributed with conception, design, interpretation of data and revision of the manuscript. All authors read and approved the final manuscript.

## Supplementary Material

Additional File 1 Brazilian Child-OIDP. Instructions, questionnaire and record form of the Brazilian Child-OIDP index. Click here for file
