# Supplementary material for: Child-OIDP index in Brazil: Cross-cultural adaptation and validation
Source: Health Qual Life Outcomes. 2008 Sep 15;6:68. doi: 10.1186/1477-7525-6-68 (PMC2553067; doi:10.1186/1477-7525-6-68)
Supplement: Additional File 1 — Brazilian Child-OIDP. Instructions, questionnaire and record form of the Brazilian Child-OIDP index. [file 1477-7525-6-68-S1.pdf]

Índice de Impactos Odontológicos no Desempenho das Atividades Diárias da Criança (OIDP-Infantil)

Instruções para aplicação

Identificação

A identificação da criança deve ser preenchida antes da aplicação do questionário.

Etapas da Entrevista

1ª Etapa: Identificar os problemas bucais percebidos pelas crianças

A entrevista começa com a Folha 1, entregue a todas as crianças presentes em sala de aula, sendo que o professor deve ler as perguntas. A criança deverá marcar com um X o(s) problema(s) que tem ou teve nos últimos 3 (três) meses (dar uma referência dizendo o nome do mês em que se iniciou o período, de acordo com a data da entrevista. Ex: se a entrevista é no mês de março, dizer "desde o mês de janeiro").

2ª Etapa: Avaliação dos impactos bucais na qualidade de vida das crianças

A entrevista individual conta com o auxílio de uma lista completa dos problemas bucais (Folha 1) e a escala analógica e facial (Quadro 1).
As respostas serão anotadas no Formulário de Registro OIDP-Infantil (Folha 2).

Inicie a entrevista

Instruções para o preenchimento da Folha 1

Entregue a Folha 1 e leia para as crianças:

Isto não é prova. Não existe resposta certa ou errada. Sinta-se à vontade para responder as perguntas. Suas respostas são muito importantes porque elas ajudarão os dentistas a entenderem pessoas como você e adotarem um tratamento dentário adequado às necessidades. Portanto, pense com cuidado antes de responder o que realmente aconteceu com você. (Em seguida, leia o item "**1ª Etapa**" da Folha 1 para as crianças e aguarde que elas respondam)

Instruções para o preenchimento da Folha 2

A criança deve estar com a Folha 1 respondida em mãos. Em seguida, passe ao preenchimento da Folha 2.

Leia para as crianças:

Considerando as suas respostas sobre os problemas que você teve com seus dentes e boca nos últimos três meses, eu gostaria de saber como eles afetaram o seu dia a dia.

Nos últimos três meses, algum destes problemas, marcados na Folha 1 (aponte para a folha), causou dificuldade para ____________? (LER CADA ATIVIDADE NO FORMULÁRIO DE REGISTRO – FOLHA 2)

*Faça a pergunta para cada atividade. Se para uma atividade a resposta for **NÃO**, então marque **0 (ZERO)** tanto para gravidade como também para a freqüência e passe para a próxima atividade. Se a resposta for **SIM**, leia as Perguntas: a) Gravidade, b) Freqüência e c) Problemas Percebidos, para cada atividade.*

Pergunta a) Gravidade

Vou fazer algumas perguntas sobre a gravidade desta dificuldade quando ela aconteceu.

Observe a escala abaixo de 1 a 3. A gravidade da dificuldade aumenta da esquerda para a direita da escala. A figura 1 representa **pequena dificuldade**, a figura 2 representa **média dificuldade** e a figura 3 representa **grande dificuldade** (Usar a escala do quadro 1).

Qual número você acha que reflete o efeito da dificuldade para ___________ (LER A ATIVIDADE) no seu dia a dia?

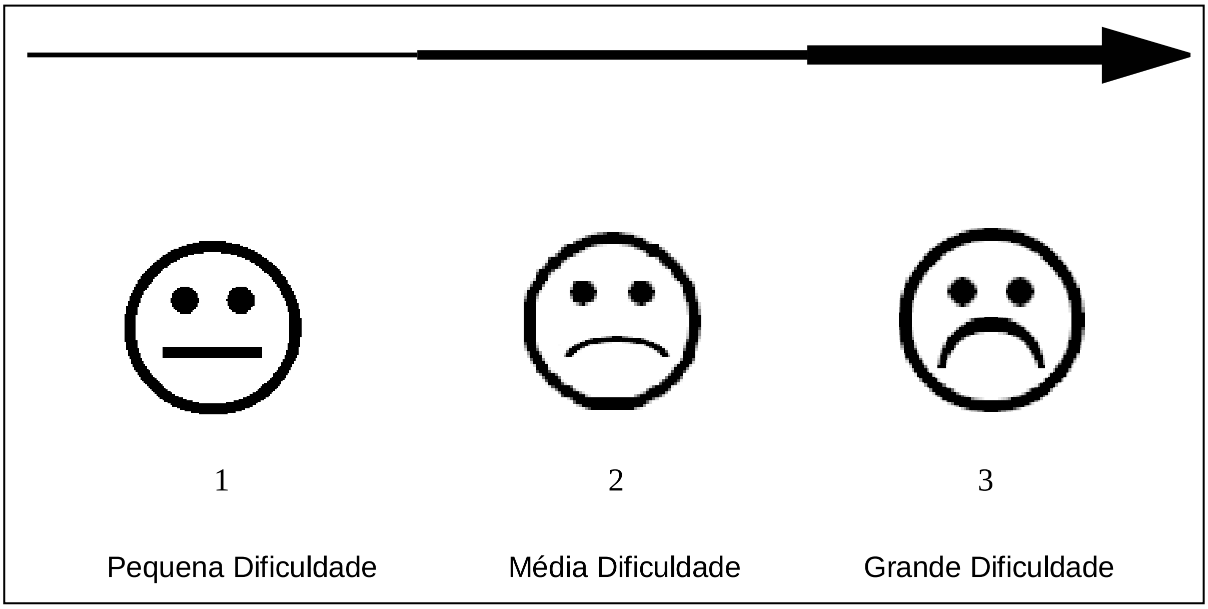

Quadro 1 - Escala analógica e facial

Pergunta b) Freqüência

Aconteceu uma ou mais vezes por mês ou menos de uma vez por mês?

1- Se aconteceu uma ou mais vezes por mês, siga para a Pergunta b1

2- Se aconteceu menos de uma vez por mês, siga para a Pergunta b2

Pergunta b1) Se aconteceu uma ou mais vezes por mês:	
- Uma ou duas vezes por mês	1
- Três ou mais vezes ao mês ou uma ou duas vezes por semana.	2
- Três ou mais vezes por semana	3

Pergunta b2) Se a dificuldade aconteceu menos de uma vez por mês, por quantos dias aconteceu no total?	
- 1 a 7 dias	1
- 8 a 15 dias	2
- mais do que 15 dias	3

Pergunta c) Problemas Percebidos

Nesse momento a criança deve ter a Folha 1 em mãos e cada atividade do Formulário de Registro deve ser lida e relacionada com os problemas marcados. Escreva na coluna "Problema(s)" o(s) código(s) do(s) problema(s) assinalado(s) pela criança na Folha 1, que tenha(m) afetado a "Atividade" da linha correspondente.

Por exemplo: se a atividade "Comer (ex. refeição, sorvete)" estiver sendo dificultada por "Dor de dente", "Cárie ou buraco no dente" e "Dente de leite mole" devem ser marcados os códigos 1, 3 e 4 na coluna "Problema(s)".

Observação: se a resposta da Folha 1 incluir "Outros", escreva o(s) problema(s) por extenso.

De acordo com os problemas de sua boca e de seus dentes, listados na Folha 1 (da 1ª Etapa), você pode dizer qual(is) deles causou (causaram) dificuldade para ____________ ? (LER CADA ATIVIDADE NO FORMULÁRIO DE REGISTRO - FOLHA 2)

1- Dor de dente
2- Dentes sensíveis (quando come ou bebe: doces, alimentos quentes como leite ou café e gelados como sorvete)
3- Cárie ou buraco no dente
4- Dente de leite mole
5- Espaço entre os dentes (porque um dente permanente, novo ou definitivo, não nasceu)
6- Dente permanente (novo ou definitivo) quebrado
7- Cor do dente (ex. mais amarelado, escuro ou manchado)
8- Forma ou tamanho do dente (ex. dente com forma ou tamanho anormal, maior ou menor que os outros)
9- Posição do dente (ex. dentes encavalados, trepados, tortos, para fora ou separados)
10- Sangramento na gengiva (ex. quando escova os dentes)
11- Gengiva inchada (ex. inflamada, ou mais avermelhada)
12- Tártaro
13- Feridas na boca
14- Mau hálito (bafo)
15- Boca ou rosto deformados (ex. lábio leporino, fenda palatina)
16- Dente permanente (novo ou definitivo) nascendo
17- Dente permanente (novo ou definitivo) perdido, faltando ou arrancado
Outros Quais? ____

Quadro 2 - Códigos da lista de problemas bucais preenchida na 1ª Etapa

FOLHA 1

Índice de Impactos Odontológicos no Desempenho das Atividades Diárias da Criança (OIDP-Infantil)

Número do termo de consentimento: ________ Data da aplicação: ______ / _____ / _______

Escola:_______________________________________ Turma: _______ Idade:____ anos

Nome: ____

Data de nascimento: ______/______/_______ Sexo: ( ) masculino ( ) feminino

1ª Etapa: Nos últimos três meses, desde ____________, você teve algum problema na sua boca ou nos seus dentes?

Marque com um X o(s) problema(s) que você tem ou teve nos últimos 3 meses.

( ) Dor de dente
( ) Dentes sensíveis (quando come ou bebe: doces, alimentos quentes como leite ou café e gelados como sorvete)
( ) Cárie ou buraco no dente
( ) Dente de leite mole
( ) Espaço entre os dentes (porque um dente permanente, novo ou definitivo, não nasceu)
( ) Dente permanente (novo ou definitivo) quebrado
( ) Cor do dente (ex. mais amarelado, escuro ou manchado)
( ) Forma ou tamanho do dente (ex. dente com forma ou tamanho anormal, maior ou menor que os outros)
( ) Posição do dente (ex. dentes encavalados, trepados, tortos, para fora ou separados)
( ) Sangramento na gengiva (ex. quando escova os dentes)
( ) Gengiva inchada (ex. inflamada, ou mais avermelhada)
( ) Tártaro
( ) Feridas na boca
( ) Mau hálito (bafo)
( ) Boca ou rosto deformados (ex. lábio leporino, fenda palatina)
( ) Dente permanente (novo ou definitivo) nascendo
( ) Dente permanente (novo ou definitivo) perdido, faltando ou arrancado
( ) Outros Quais? ____

FOLHA 2

Formulário de Registro OIDP-Infantil

Atividades	a)Gravidade	b)Freqüência		c)Problema(s)
		b1	b2	
1. Comer (ex. refeição, sorvete)				
2. Falar claramente				
3. Limpar a boca (ex. escovar os dentes e bochechar)				
4. Dormir				
5. Manter o seu estado emocional (humor) sem se irritar ou estressar				
6. Sorrir, rir e mostrar os dentes sem sentir vergonha				
7. Fazer as tarefas da escola (ex. ir à escola, aprender em sala de aula, fazer o dever de casa)				
8. Ter contato com as pessoas (ex. sair com amigos, ir à casa de um amigo)				
